# Intensity of contact with frontline workers and its influence on maternal and newborn health behaviors: cross-sectional survey in rural Uttar Pradesh, India

**DOI:** 10.1186/s41043-017-0129-6

**Published:** 2018-01-08

**Authors:** Tanica Lyngdoh, Sutapa B. Neogi, Danish Ahmad, Srinivasan Soundararajan, Dileep Mavalankar

**Affiliations:** 1 Indian Institute of Public Health Delhi, Public Health foundation of India, New Delhi, India; 2 Public Health foundation of India, New Delhi, India; 3 Indian Institute of Public Health Gandhinagar, Public Health Foundation of India, New Delhi, India; 4 Indian Institute of Public Health Delhi, Public Health foundation of India, Plot No. 47, Sector-44, Institutional Area, Gurgaon, 122002 India

## Abstract

**Background:**

India is committed to improving maternal and newborn health in order to achieve the targets for India’s Millennium Development Goal 4. Considering their role as a link between community and health systems, frontline workers (FLWs) can be effectively utilized in strengthening maternal and newborn care. In this paper, we set out to examine the effect of intensity of contact with FLWs on key maternal and newborn health behaviors and to determine if this association varies by status of Self Help Group (SHG) membership.

**Methods:**

This population-based cross-sectional study included 2208 currently married women aged 15–49 years who had delivered a baby during the last 15 months prior to the survey and selected through a multi-stage cluster sampling from rural villages and urban wards. The outcome of interest included variables related to key knowledge and practice of healthy behavior in relation to maternal and newborn health and exposure variable considered was intensity of contact with FLWs.

**Results:**

Of the women interviewed, 1729 (78%) belonged to SHG household. For knowledge on the need for at least 3 antenatal care (ANC) check-ups, two tetanus toxoid (TT) injections and consumption of 100 or more iron-folic acid (IFA) tablets, proportion of those who were aware of these practices increased with increasing number of contacts with FLWs (*P* value < 0.001). Practice for TT injections showed an increasing trend with increasing number of contacts with FLW. An increase in the odds of delivering in an institution was observed in those who had higher number of contacts as compared to those with no contacts (*P* value < 0.001). With regard to newborn healthy behavior practice, breastfeeding within 1 h of delivery showed significant association and the odds of this practice improved in those who had ≥ 3 contacts with FLW as compared to those had no contacts. Except for consumption of 100 or more IFA tablets, there was no interaction of these associations by SHG status.

**Conclusion:**

There was an overall low prevalence of both knowledge and practice of key maternal and newborn healthy behaviors and only a few of these were associated with frequency of contacts with FLW. Findings not only highlight the urgent need for effectively leveraging FLWs to strengthen maternal and newborn care but also to improve the quality of services provided by them.

**Electronic supplementary material:**

The online version of this article (10.1186/s41043-017-0129-6) contains supplementary material, which is available to authorized users.

## Background

Despite the commitment and concerted effort in adopting the framework laid down under the Millennium Development Goals (MDGs), India has failed to achieve the targets, in particular, reduction in Maternal Mortality Ratio (MMR) and Infant Mortality Rate (IMR) [1, 2]. Post 2015, the Sustainable Development Goals (SDGs) have set the agenda for prioritizing maternal and newborn health by applying lessons learnt from the MDGs and are working towards developing strategies that will achieve a steeper decline. Aggressive steps at this point are crucial to accelerate the progress in reducing maternal and newborn mortality in order to meet the SDG targets.

The health of mothers and newborns are closely related and interventions to prevent mortality essentially apply to both. It is well known that improvements in maternal and newborn health does not necessarily require high-tech, complex interventions. Even though simple measures like antenatal care, clean birth practices, essential newborn care, early initiation, and exclusive breastfeeding can go a long way to bring about positive impact, yet gaps persist in the practice of good maternal and newborn health behaviors. Globally, there is increasing interest in the role of frontline workers (FLW) as an effective tool in bridging this gap. Community-based strategies involving the expanded use of FLWs as a link to health system strengthening for maternal and newborn care have proven successful as evident from a community-based trial in Uganda [3]. Further, Adam et al. found delivery of health messages by community health workers increased knowledge of maternal and newborn care among women in the local community [4]. A recent systematic review by Gilmore and colleagues suggest that FLWs are being widely utilized in middle and low income countries and there is evidence (albeit insufficient) to suggest that they could be an effective tool in reducing maternal and neonatal mortality [5]. Hence, in resource-constrained settings like ours, there is a huge potential for the role of community health workers in improving maternal and newborn care.

In India, FLWs (including Auxiliary Nurse Midwives (ANMs), Accredited Social Health Activists (ASHAs), and Anganwadi worker (AWWs)) form an integral part of the Indian healthcare system and are key elements of delivery of healthcare services in the community. They are the links between the community and the healthcare system and often first point of contact for people seeking care. The FLWs function as service extension workers and as activists for social change and play a pivotal role by supporting programs to improve access to healthcare and nutrition. They are female cadres of India’s community health worker program selected from the community itself which helps in local and cultural acceptability of their roles by the community.

Presently, there is little data available in the Indian context that documents the frequency of contact with FLWs in promoting maternal and newborn related healthy behavior. Thus, the current analysis was undertaken to examine the effect of intensity of contact with FLWs on maternal and newborn health behaviors and to determine if the association varied by status of Self Help Group (SHG) membership. Uttar Pradesh (UP), the most populous state of India with high levels of poverty and low levels of social and economic development [6], has received unprecedented attention and strong political commitment from the state as well as national government in efforts aimed at improving maternal and child health and reducing related mortality. Yet, it still continues to perform poorly and contributes to 28 and 35% of neonatal and maternal deaths in the country [7]. As per the Sample Registration Survey 2013 (SRS, 2013), the estimates of MMR and IMR of UP are at 292 per 100,000 live births and 50 per 1000 live births respectively. Current analyses are performed using data from the baseline learning phase of the Uttar Pradesh Community Mobilization (UPCM) Project [8] (the details of which are described below) to better understand the experiences of integrating FLWs into health systems to improve maternal and newborn outcomes in a setting such as UP.

## Methods

### Study design and settings

The UPCM project utilized a pre-post intervention-control design. However, the current paper is an exploratory analysis that utilized data from the baseline survey of the UPCM project. The project, a 5-year initiative, started in 2011 with the aim to develop and scale up evidence-based interventions to achieve the goal of improving Reproductive, Maternal, Newborn and Child Health and Nutrition (RMNCHN) health behaviors among marginalized population in UP. The goal of this l project was to reduce neonatal mortality by improving maternal and child health behaviors through developing and scaling up a package of family health interventions and strengthening the management of Behavior Change Communication (BCC) using SHGs as a platform. In UP, the Rajiv Gandhi Mahila Vikas Pariyojana (RGMVP) working since 2002 developed a rapidly scalable SHG model organizing poor rural women into groups of 10–20 women and using them as platforms for building up family health interventions. All the SHGs mobilized at the village level are federated into village organizations (VOs) representing 150–250 women drawn from 10 to 20 SHGs. These VOs, in turn, are federated into Block Organizations (BOs) representing 5000–7000 women. The RGMVP program utilizes community health volunteers (called Swasthya Sakhi, SS) who are selected from among the members of SHG (1 SS per SHG). She is trained to counsel women and their families on key health behaviors. The SHG acts as a systemic interface between poor people and development initiatives. The RGMVP is an independent platform and is not directly linked to the existing healthcare delivery system.

The UPCM program under which the baseline survey was conducted, utilized the SSs and mobilized the community using the SHG platform. This social platform served as a vehicle for behavior change management (BCM) as well as for improving health system access through collaboration with local level field workers. The UPCM project was not directly linked to the existing government health system; it was not involved in delivery of health services per se.

The baseline survey of the UPCM project was carried out in 5 districts (Sultanpur, Raebareli, Mirzapur, Maharajganj and Hardoi) in UP from June to August 2013. The districts were purposively selected based on geographical diversity and the presence of the SHGs. The current analyses utilized the overall data from baseline survey.

### Sampling and study population

A two-staged sampling was used to first select districts and then required number of households (Please refer to Additional file 1: Box S1 for an overview of administrative division in India). In each block of the five selected districts, a house listing was done in all villages/*purvas* (group of SHGs) of the listed GPs to identify eligible women among SHG households. The house listing included the name of the household’s head, the woman’s name and the age of her last child during the survey. A complete enumeration of the listed eligible women was done to identify women from SHG households in intervention and control areas. Selection of respondents was done through availability of eligible woman at the time of sample collection. To identify eligible women from non-SHG households in the intervention area, a listing of non-SHG households was done in the neighborhood of SHG households in each selected village/*purva* to ensure similar socioeconomic characteristics of women in both the groups. The required number of non-SHG households (half of the sampled SHG households) was randomly selected from each village/*purva*. The eligibility criteria for inclusion into the sampling frame were (a) currently married women aged 15 to 49 years and (b) who had delivered a baby till 15 months prior to the survey. If there was more than one eligible woman per household, a random procedure was used to select the woman. The sampling strategy used was in line with the objectives of the baseline survey, that is, to establish equivalence of intervention and control areas with respect to background characteristics and to assess baseline level of maternal and child health practices between the two arms. However, for the purpose of this paper, the data from the overall sample was analyzed without differentiating it into intervention and control arms.

### Sample size

Among the different indicators related to health behavior, change in percentage points of institutional delivery was used for calculating the sample size as it produced the largest sample size. A sample of 878 eligible women from SHG households was required in each arm (intervention and control) assuming a 20 percentage point increase in institutional delivery over 3 years with 80% power, (5% confidence and 10% non-response rate. Additionally, to assess the diffusion effect in adoption of healthy behaviors, half the number (439) was required in the intervention area from households without SHG member. Thus, the total sample size required was 2195 eligible women (878 + 878 + 439). However, a few additional women were interviewed and the final sample included 2208 women. This sample size calculation was meant to answer the main objective of UPCM project, that is, to measure the effect of BCC intervention on healthy maternal and newborn behavior changes. The current analysis is only an exploratory study and used the overall data from the baseline learning survey.

### Study tool and measurements

The baseline survey was conducted by the Population Council, one of the implementing partners and responsible for the Monitoring, Learning and Evaluation (MLE) activities of the project. Structured interviews (in Hindi) programmed on a computer-assisted personal interview (CAPI) package was orally administered to collect information using mini-laptops by specially trained field staff. Census and Survey Processing System (CSPro) software compatible with the mini-laptops was used. A series of internal consistency checks and logics were built into the CAPI package for data quality. The outcome of interest included variables related to knowledge and practice of healthy behavior in relation to maternal and newborn health. The knowledge indicators related to maternal health behavior considered for this analysis were binary responses to questions on availing at least 3 antenatal care (ANC) check-ups (those who reported this as “don’t know” or “as many as required” were considered as “No”), taking at least two Tetanus Toxoid (TT) injections, consuming 100 or more Iron-Folic Acid (IFA) tablets, awareness of key danger signs during pregnancy, delivery and after delivery for which immediate attention was required; knowledge on key newborn health behavior included responses to questions on knowledge that nothing needs to be applied to cord stump, that breastfeeding needs to be initiated within 1 h following delivery and knowledge on how Kangaroo Mother care (KMC)/skin-to-skin is to be done. Practice of maternal health behavior was captured through key indicators like attended at least 3 ANCs, took at least two TT injections, consumed 100 or more IFA tablets, saved money for delivery, arranged transport for delivery, decided place of delivery, delivered at institution, sought treatment for complications during delivery and within 42 days after delivery and received any post-natal care (PNC) within 42 days of delivery. Key indicators on practice of newborn health behavior considered for this analysis were seeking treatment for danger signs experienced by newborn during first month of birth, applying nothing on cord stump, delaying bath till after 24 h of delivery, practicing KMC/skin-to-skin, breastfeeding within 1 h of delivery, and feeding mother’s yellow thick milk to child.

FLW provides services in close proximity to the community [9]. These services include those that are delivered at home or family and through outreach sessions. These can be further categorized into services offered during pregnancy, delivery, and during neonatal period. In India, female health workers or ANMs take care of a population of 5000. ASHAs and AWWs are responsible for a population of 1000 each. SS. The SSs under the RGMVP program are mainly involved in facilitating awareness and ensuring women’s access to healthcare services. Please refer to Additional file 1: Box S1 for further details on the different FLWs and their links with healthcare system.

For the purpose of this paper, the term “FLW” refers to community health workers under the Indian healthcare system including ANMs, ASHAs and AWWs and the SSs who are community volunteers under the RGMVP program. The exposure variable considered was intensity of contact with FLWs during pregnancy (measured as total number of contacts with either ANMs, ASHAs, AWWs or SSs). This was further arbitrarily categorized into three groups—no contact, 1–2 contacts, and ≥ 3 contacts. In addition, information on other potential confounding variables like age, highest standard of education completed by women, if women presently working, religion of head of the family, caste of head of the family (a system of social stratification in India dividing Hindus into hierarchical groups; those belonging to scheduled caste, scheduled tribe and other backward classes are among the lower caste groups), wealth index, and history of previous stillbirth were collected.

### Statistical analysis

All tests were performed using Stata 13 (StataCorp, College Station, TX, USA). Categorical variables as number of subjects and percentages while continuous variables were summarized as mean ± standard deviation (SD). We used chi-square test to compare the differences in the distribution of categorical variables across groups that summarized frequency of visits to FLW. We examined the association of intensity of contact with FLW (independent variable of interest) with maternal and newborn health behavior practice indicators as the dependent variable, one at a time using regression models. Multilevel simple and multiple logistic regression with random intercept was used for analyses to account for the complex sampling design. We started by univariate analysis and subsequently fitted multivariable model adjusting for age, highest standard of education completed by women, if women presently working, religion of head of the family, caste of head of the family, wealth index, and history of previous stillbirth. To assess if the relation was modified by SHG membership, we included a multiplicative interaction parameter between SHG membership status and each of the practice indicators into the regression models. The significance level used for two-sided tests was *P* < 0.05.

#### Ethics

The project proposal was reviewed and approved by Independent Ethics Committee of Population Council, New Delhi. Verbal consent for participation was obtained from the participants by the field interviewers before collecting the data.

## Results

Analysis was done on a total of 2208 women, of whom 78% belonged to SHG household. Mean age of these women was 25.55 ± 4.92 years with only 52% having ever attended school and about 20% currently engaged in any form of work to earn either in cash/kind or both. The mean number of living children these women had was 2.60 ± 1.56 and about 6% of them reported pregnancies resulting in a stillbirth. Majority of them (93%) were Hindus predominantly belonging to scheduled tribe (6%), scheduled caste (47%), or other backward class (35%) (data not shown).

Table 1 describes women’s contact with different frontline workers. The results show that during pregnancy, about 91% of women had some contact with at least one of the FLWs of whom 14% had 1–2 contacts and 77% had 3 or more contacts. Among those who had some contact, 81% reported contact with ANM; 78% with ASHA; 35% with AWW; and about 2% with SS. The average number of contacts with ANM, ASHA, AWW and SS were 2.36, 3.02, 3.11, and 2.86, respectively.

**Table 1:** Contact with frontline worker (FLW) and intensity of contact

Contacts with FLW	n (%) out of 2208	Average number of contacts
ANM	1791(81.11)	2.36
ASHA	1724 (78.08)	3.02
AWW	780 (35.33)	3.11
SS	42 (1.90)	2.86

*ANM* auxiliary nurse midwives, *ASHA* accredited social health activist, *AWW* Anganwadi worker, *SS* Swasthya Sakhi

Table 2 describes the distribution of key socio-demographic characteristics of the women across categories reflecting intensity of contact. All socio-demographic characteristics considered in this analysis did not differ significantly with intensity of contact with FLWs except for presently working which showed a weak evidence of a difference. Distribution of knowledge and practice of key maternal healthy behaviors among women across categories summarizing intensity of contact is presented in Table 3. Over 50% women had knowledge that one must undergo at least 3 ANC check-ups, 57% were aware of the need of two TT injections but less than one-third had knowledge about the need for consumption of 100 or more IFA tablets. For all these three maternal behaviors, knowledge showed a significant increasing trend with an increasing number of contacts (*P* value < 0.001). Knowledge of danger signs/complications during pregnancy, delivery, and post-partum period requiring immediate attention was varied and ranged from < 1% for convulsions to about 20% for excessive vaginal bleeding during pregnancy and post-partum period (data not shown). With regard to practice of healthy maternal behaviors, 96% of women reported having taken TT injections as a part of ANC check-ups during their last pregnancy. However, uptake of ANC check-ups and consumption of 100 or more IFA tablets were only 51 and 8% respectively even though awareness about them was slightly higher. Except for TT injections which showed an increasing trend with increasing number of contacts with FLW, there were no significant associations of ANC check-ups and IFA consumption with intensity of contact with FLW. Further, no associations were observed between delivery preparedness (comprising of saving money, arranging transport, deciding place of delivery and identifying the institution in case of emergency), seeking treatment for complications during delivery and within 42 days after delivery with intensity of contact with FLWs. Only 68% of these women reported having delivered their last child at an institution and the proportion increased with increasing number of contact with FLWs (*P* value < 0.001).

**Table 2:** Distribution of key demographic characteristics according to intensity of contact with FLW

	No. of contact with FLW			
	0 (*N* = 202)	1–2 (*N* = 316)	≥ 3 (*N* = 1690)	*P* value
Mean age of women in years (SD)	25.15 (5.50)	24.99 (4.48)	25.70 (4.92)	0.100
Mean number of living children (SD)				
Highest educational standard attained, *n*(%)	95 (47.03)	172 (54.43)	888 (52.54)	0.428
No education	107 (52.97)	145 (45.89)	808 (47.81)	
Up to 10th standard	71 (35.15)	137 (43.35)	703 (41.60)	
Above 10th standard^a^	24 (11.88)	34 (10.76)	179 (10.59)	
Working for cash or kind, n (%)	35 (17.33)	54 (17.09)	368 (21.78)	0.078
Religion, *n*(%)				
Hindu	186 (92.08)	285 (90.19)	1575 (93.20)	0.161
Muslim	16 (7.92)	31 (9.81)	115 (6.80)	
Caste, *n*(%)				
Scheduled caste/scheduled tribe	113 (55.94)	162 (51.27)	897 (53.08)	0.822
Other backward classes	69 (34.16)	114 (36.08)	589 (34.85)	
Others	20 (9.90)	40 (12.66)	204 (12.07)	
Wealth Index, *n*(%)				
Highest	28 (13.86)	76 (24.05)	338 (20.00)	0.208
Second	42 (20.79)	62 (19.62)	338 (20.00)	
Third	45 (22.28)	62 (19.62)	334 (19.76)	
Fourth and fifth quintile combined	87 (43.07)	116 (36.71)	680 (40.24)	
Type of family, *n*(%)				
Nuclear	85 (42.08)	123 (38.92)	784 (46.39)	0.035
Non-nuclear	117 (57.92)	193 (61.08)	906 (53.61)	
History of previous stillbirth	17 (8.42)	18 (5.70)	103 (6.09)	0.396

^a^This category includes 11th and12th standard, Bachelors, Post-graduates, Technical education after 10th standard and others combined

**Table 3:** Distribution of key maternal knowledge and practice health indicators during pregnancy, delivery and after pregnancy according to intensity of contact with FLW

		No. of contact with FLW				
	Overall (*N* = 2208)	0 (*N* = 202)	1–2 (*N* = 316)	≥ 3 (*N* = 1690)	*P* value	*P* value for trend
Knowledge						
At least 3 ANC	1232 (55.80)	75 (37.13)	152 (48.10)	1005 (59.47)	< 0.001	< 0.001
Taking two TT injections	1250 (56.61)	90 (44.55)	179 (56.65)	981 (58.05)	0.001	0.001
Consuming 100 or more IFA tablets	624 (28.26)	37 (18.32)	73 (23.10)	514 (30.41)	< 0.001	< 0.001
Practice						
At least 3 ANC	1492 (67.57)	163 (80.69)	175 (55.38)	1154 (68.28)	< 0.001	0.300
Consumed 100 or more IFA tablets	175 (7.83)	16 (7.92)	28 (8.86)	131 (7.75)	0.799	0.716
Taken TT injection	1770 (96.09)	89 (81.65)	241 (93.77)	1440 (97.56)	< 0.001	< 0.001
Saved money for delivery	817 (27.10)	54 (29.51)	92 (27.22)	671 (26.90)	0.745	0.490
Arranged transport for delivery	380 (12.60)	19 (10.38)	41 (12.13)	320 (12.83)	0.605	0.330
Decided place of delivery	289 (9.59)	23 (12.57)	46 (13.61)	220 (8.82)	0.007	0.006
Identified institution in case of emergency	53 (1.76)	2 (1.09)	2 (0.59)	49 (1.96)	0.154	0.107
Delivered at institution	1496 (67.71)	111 (54.95)	186 (58.86)	1198 (70.89)	< 0.001	< 0.001
Sought treatment for complications during delivery^a^	859 (85.64)	66 (82.50)	99 (83.90)	694 (86.21)	0.564	0.288
Sought treatment for complications within 42 days after delivery^a^	812 (87.97)	63 (86.30)	98 (85.96)	651 (88.45)	0.675	0.425
Received any PNC within 42 days of delivery	580 (26.27)	50 (24.75)	69 (21.84)	461 (27.28)	0.114	0.125

*FLW* frontline worker, *ANC* antenatal care, *TT* tetanus toxoid, *IFA* iron-folic acid, *PNC* postnatal care

^a^The denominator used were women reporting problems/complications and not the total sample *N*

Knowledge of key newborn healthy behaviors was limited to the awareness that breastfeeding should be initiated within 1 h (Table 4) of delivery while awareness on thermal care (KMC/skin-to-skin) and knowledge that nothing is to be applied to the cord stump was very poor (about 3%). Practice of breastfeeding within 1 h of delivery was observed in 50% of the women. Similarly, 56% of women reported delayed bath of newborn till after 24 h of delivery. Both practices showed increasing trends with increasing number of contacts with FLW.

**Table 4:** Distribution of key newborn knowledge and practice health indicators according to intensity of contact with FLW

		No. of contact with FLW				
	Overall (*N* = 2208)	0 (*N* = 202)	1–2 (*N* = 316)	≥ 3 (*N* = 1690)	*P* value	*P* value for trend
Knowledge						
Knowledge that nothing needs to be applied to cord stump	93 (3.40)	5 (2.02)	12 (3.13)	76 (3.61)	0.407	0.190
Knowledge that breastfeeding should be initiated within 1 h	1285 (58.20)	93 (46.04)	171 (54.11)	1021 (60.41)	< 0.001	< 0.001
Know how KMC/skin-to-skin care is done	49 (3.42)	3 (2.46)	3 (1.65)	43 (3.81)	0.275	0.190
Practice						
Sought treatment for danger signs experienced by newborn during first month of birth^a^	2041 (84.48)	145 (82.39)	285 (84.82)	1611 (84.61)	0.725	0.555
Applied nothing on cord stump	601 (23.85)	52 (22.71)	89 (24.86)	460 (23.80)	0.832	0.905
Delayed bath (after 24 h)	1255 (56.84)	96 (47.52)	167 (52.85)	992 (58.70)	0.003	0.001
Practice KMC/skin-to-skin	59 (3.31)	2 (1.39)	5 (2.30)	52 (3.66)	0.237	0.091
Breastfeeding within 1 h	1072 (49.52)	70 (36.46)	129 (41.48)	873 (52.53)	< 0.001	< 0.001
Fed mother’s yellow thick milk to child	1836 (83.15)	155 (76.73)	265 (83.86)	1416 (83.79)	0.038	0.034

*FLW* frontline worker, *KMC* Kangaroo Mother Care

^a^The denominator used were new born children reporting danger signs

Tables 5 and 6 describe the crude and adjusted associations of practice of maternal and newborn healthy behavior respectively with intensity of contact with FLW. The odds of taking TT injection was 5 and 6 times more in those who had 1–2 and ≥ 3 contacts respectively as compared to those with no contacts (*P* value < 0.001). However, no such association was observed with the uptake of at least 3 ANCs. A similar increase in odds of delivering in an institution was observed in those who had higher number of contacts as compared to those with no contacts (*P* value < 0.001). With regard to newborn healthy behavior practice, breastfeeding within 1 h of delivery showed significant association and the odds of this practice improved in those who had ≥ 3 contacts with FLW as compared to those had no contacts. Except for consumption of 100 or more IFA tablets, we did not find evidence of any interaction of these associations by SHG status, suggesting that associations between contact with FLW and healthy behavior did not significantly differ by SHG membership (that is, there was no additional benefit of being an SHG member).

**Table 5:** Crude and adjusted associations of practice of maternal healthy behavior with intensity of contact with FLW

	No. of contact with FLW							
	0 (*N* = 202)	1–2 (*N* = 316)		≥ 3 (*N* = 1690)		*P* value		*P* value for interaction
Practice indicators	Ref	Crude OR (95%CI)	Adjusted (95%CI)	Crude (95%CI)	Adjusted (95%CI)	Crude	Adjusted	
At least 3 ANC check-ups	[1]	0.28 (0.18–0.42)	0.29 (0.16–0.53)	0.47 (0.32–0.68)	0.63 (0.37–1.08)	< 0.001	< 0.001	0.751
Consumed 100 or more IFA tablets	[1]	1.06 (0.55–2.01)	0.88 (0.40–1.95)	0.84 (0.48–1.46)	0.72 (0.37–1.41)	0.537	0.538	0.018
Taken TT injection	[1]	4.15 (2.76–6.23)	4.52 (2.36–8.65)	7.03 (5.00–9.88)	5.86 (3.50–9.82)	< 0.001	< 0.001	0.739
Saved money for delivery	[1]	0.89 (0.60–1.34)	0.93 (0.54–1.59)	0.90 (0.64–1.25)	0.84 (0.54–1.31)	0.816	0.674	0.348
Arranged transport for delivery	[1]	1.16 (0.65–2.08)	1.25 (0.58–2.71)	1.27 (0.77–2.10)	1.35 (0.70–2.60)	0.580	0.662	0.963
Decided place of delivery	[1]	1.15 (0.66–2.01)	1.02 (0.47–2.23)	0.78 (0.48–1.26)	0.81 (0.42–1.55)	0.078	0.566	0.462
Delivered at institution	[1]	1.04 (0.70–1.55)	0.67 (0.35–1.29)	1.61 (1.15–2.25)	1.10 (0.62–1.94)	0.001	0.064	0.252
Sought treatment for complications during delivery	[1]	1.21 (0.50–2.95)	1.10 (0.31–3.95)	1.23 (0.59–2.56)	1.97 (0.67–5.77)	0.857	0.236	0.500
Sought treatment for complications within 42 days after delivery	[1]	0.97 (0.39–2.44)	1.23 (0.16–9.45)	1.14 (0.53–2.44)	0.35 (0.07–1.79)	0.863	0.102	0.113
Received any PNC within 42 days of delivery	[1]	0.80 (0.51–1.25)	0.76 (0.42–1.38)	0.93 (0.64–1.35)	0.87 (0.53–1.44)	0.542	0.659	0.888

*FLW* frontline worker, *ANC* antenatal care, *TT* tetanus toxoid, *IFA* iron-folic acid, *PNC* postnatal care

**Table 6:** Crude and adjusted associations of practice of newborn health behavior with intensity of contact with FLW

	No. of contact with FLW							
	0 (*N* = 202)	1–2 (*N* = 316)		≥ 3 (*N* = 1690)		*P* value		*P* value for interaction
Practice indicators	Ref	Crude OR (95%CI)	Adjusted (95%CI)	Crude (95%CI)	Adjusted (95%CI)	Crude	Adjusted	
Sought treatment for danger signs experienced by newborn during first month of birth	[1]	1.26 (0.75–2.11)	1.00 (0.46–2.17)	1.21 (0.78–1.87)	1.09 (0.56–2.12)	0.648	0.925	0.133
Applied nothing on cord stump	[1]	1.20 (0.80–1.82)	1.36 (0.73–2.54)	1.02 (0.72–1.46)	1.56 (0.91–2.67)	0.508	0.242	0.659
Delayed bath (after 24 h)	[1]	1.19 (0.82–1.72)	0.99 (0.56–1.73)	1.32 (0.97–1.81)	1.13 (0.70–1.83)	0.182	0.714	0.801
Practice KMC/skin-to-skin	[1]	1.69 (0.32–9.03)	1.17 (0.20–6.87)	2.55 (0.60–10.82)	1.25 (0.27–5.77)	0.334	0.957	
Breastfeeding within 1 h	[1]	1.11 (0.76–1.63)	0.78 (0.45–1.33)	1.67 (1.21–2.31)	1.29 (0.82–2.04)	< 0.001	0.013	0.784
Fed mother’s yellow thick milk to child	[1]	1.49 (0.94–2.35)	0.58 (0.25–1.33)	1.38 (0.95–1.99)	0.74 (0.35–1.56)	0.180	0.390	0.582

*FLW* frontline worker, *KMC* Kangaroo Mother Care

## Discussion

In this baseline survey of currently married women aged 15 to 49 years predominantly Hindus belonging to scheduled tribe/scheduled caste or other backward classes, we observed an overall low prevalence of both knowledge and practice of key maternal and newborn healthy behaviors. IFA consumption, one of the key maternity care component determining maternal health, was discouragingly low among this sample of women. It is difficult to decipher (in the absence of supporting data) if observed low prevalence is because of gaps in the supply chain or the fact that pregnant women are reluctant to take it. However, recent evidence from another part of rural India suggest that not only socio-demographic determinants but ANC factors like timing, frequency, and quality play a key role in facilitating consumption [10]. The same study observed more likelihood of women consuming IFA if it is available in stock on important village days like Village Health and Nutrition Day (VHND).

Very low knowledge on newborn healthy practices like care of cord stump and KMC/skin-to-skin care was reported. This could be a reflection of the fact that although these practices have traditionally been a part of intervention initiatives for child survival but guidelines and policies under the National program were not in place until the recent years [11] and may not have been widely implemented. Further, there was surprisingly high practice of cord care in spite of very low knowledge on the same. This is difficult to explain; but a probable explanation could be that because all information was collected based on recall it is likely that recall for practice of a healthy behavior was easier as compared to that for knowledge.

Practice of institutional delivery, TT injection, and breastfeeding within 1 h of delivery was significantly associated with contact with FLW and an increasing trend was observed with increasing intensity of contact with FLW. Except for relationship between IFA consumption and contact with FLW, there was no significant interaction by SHG membership status for any of the other associations.

Among the key maternal health behaviors studied, only knowledge on the need for ≥ 3 ANCs and TT injection were significantly associated with contact with FLWs. In spite of the recommended 3 (and more recently 4) ANC check-ups, uptake of minimum required ANC visits remains low [12, 13]. Women perceive ANC care as unnecessary [14] and pregnancy as a natural phenomenon not requiring special care [15]. Uptake of ANCs check-ups in the current analysis was observed to have reduced with increasing number of contacts with FLWs. This can probably be attributed to the fact that any contact with FLW is being utilized by women for ANC counseling /check-ups and that the women did not feel the need to attend antenatal clinics in a health facility for the same if they already had some contact with a FLW. Although antenatal care is one of the strongest predictors of institutional delivery [16], we observed a high prevalence of the same in the study population in spite of low ANC coverage. The presence of an association between institutional delivery and TT injection with intensity of contact with FLW is not surprising given that maternal and child health related programs in India have been aggressively promoting institutionalization of deliveries and universal immunization of pregnant women against tetanus. Additionally, contact with FLWs probably helps to further reinforce the message and motivate the women.

The current study revealed that increasing the number of contacts with FLWs did not have any effect on knowledge of essential newborn care, in particular, cord care and thermal care but had a significant effect on initiation of breast feeding within an hour. Although the average number of contacts with different FLWs was close to 3 yet these interactions were not effectively advancing healthy behavior among the women and a commensurate improvement in awareness and behavior change towards maternal and newborn care. This could be a reflection of the fact that intensity of contact with FLWs alone does not necessarily determine healthy practices but may largely be driven by quality of services provided by FLWs. The success of delivery of maternal and newborn health care services depends largely on adequate training of FLWs; sound knowledge and good quality of services rendered by FLWs can materialize to better awareness and healthy practices in the community. Indeed, there are evidences to suggest that knowledge and practices of FLW on maternal health are far from adequate and there is a need for regular re-training and evaluation [17, 18].

With regard to the number of contacts with a FLW, it has been shown that contact within first 48 h of delivery has the greatest impact on Neonatal Mortality Rate (NMR). There is inconclusive evidence on the relationship between number of home visits and NMR. Moreover, how much would be the impact of community based interventions on NMR depends largely on the baseline NMR (more than 50 per 1000 live births) and coverage (≥ 50%) in the population [19, 20].

Globally, there is renewed interest in the engaging frontline workers in maternal and child health, particularly, in low and middle income countries where shortage of human resources necessitates their involvement to increase capacity of constrained health systems. The literature reveals evidence showing promising benefits of utilizing lay health workers in health programs, in particular, in promoting immunization and breastfeeding uptake and subsequently reducing child morbidity and mortality [21]. Other systematic reviews support these findings and have demonstrated the effectiveness of community health workers in low- and middle-income countries in delivering preventive interventions for maternal and child health [5] and preventing perinatal and maternal death by improving antenatal, perinatal and postpartum service delivery [22].

In the Indian healthcare system, FLWs play a critical role in the delivery of primary health care to promote community health and nutrition for rural communities. The FLW undergoes extensive training to dispense their roles which mainly cover preventative aspects of maternal, new-born and child care. They provide a basket of essential preventive primary care for Reproductive, Maternal, Newborn, Child, and Adolescent health (RMNCH+A) services and are linked to government health facilities where they refer pregnant woman, new-born and children for care. The ASHAs, in particular, are also incentivized under specific programs to promote maternal care i.e. promote institutional delivery and conduct postnatal care in the community. The RGMVP program in UP working for poverty reduction, women’s empowerment, and rural development, used an innovative scalable demand-side SHG model as a social platform to promote not only social and empowerment agendas but also health behavior change in select districts. While government cadre of FLWs were well established and functional in the select districts at the time of baseline survey, the saturation of the villages with the SSs under the RGMVP was still under way. The current network of FLWs under the existing government health system and the volunteers (SSs) under the RGMVP program provided a good opportunity for the UPCM project to layer the proposed health intervention. However, any observed (positive or negative) association between ANC check-ups and intensity of contacts from the present analyses is independent of the UPCM project.

Moving beyond FLWs to mobilizing community volunteers is recognized as another instrumental way that can be combined for collective action to have a sustainable impact on maternal and health outcomes. Incorporating community-based interventions through different approaches have resulted in improvement of health outcomes of mother and newborn. A recent systematic review on effect of participatory learning and action involving women’s group in low-resource setting showed how this approach can be used as a cost effective strategy to improve maternal and newborn survival [23]. Closer home, the Shivgarh trial in one district of Uttar Pradesh successfully demonstrated reduction in NMR through implementation of essential newborn care intervention package using a Behavior Change Management (BCM) approach by community mobilizers and volunteers [24]. Further, Ekjut trial in Jharkhand and Orissa in India [25] and other community mobilization efforts across neighboring countries like Bangladesh [26, 27] and Nepal [28] show evidence supporting the same. In fact, the Ekjut study demonstrated additional effects like continuation of practices post withdrawal of the trial [29]. An interesting finding in this trial was that the more marginalized benefitted most from the intervention than the less marginalized suggesting inequalities in neonatal mortality can be reduced through community-based participatory intervention [30].

Along the same lines, the potential role of social platforms like SHG in health care delivery in low- and middle-income countries has garnered attention and they have shown to complement existing health care services helping to achieve some degree of synergy between health care providers and users [31]. The current implementation project took advantage of the SHG platform initiated by RGMVP to implement their intervention involving BCM approach. At the time of the baseline survey, the RGMVP had institutionalized SHG platform in over 37 districts (including the selected districts for baseline survey) in UP with an aim to saturate villages with them. Except for IFA consumption, associations of contact with FLWs and other maternal and newborn healthy practice did not differ by SHG membership status of the women. Considering that these are findings from the baseline survey before the intervention was rolled out, it could be that although SHGs were well-established they were not adequately leveraged for promoting healthy behavior for maternal and newborn care; it is then not surprising to see no effect modification of these associations by SHG membership. Even if SHGs were existing and were functional, there is the possibility that SHG’s monthly meetings, a forum for execution of their agenda, i.e., to spread awareness among its members on health services, were not effectively being utilized. This could explain the absence of any additional effect of SHG membership on the associations between health behavior and contact with FLW.

The strengths of this study are its population-based design and reasonably large sample size. The study is limited by several factors. Assessments of maternal and newborn health indicators relied on a long recall period of 15 months and this could have an influence on the findings. While knowledge reflected awareness at time of survey, indicators of healthy behavior was assessed for practices that were followed during pregnancy, delivery, and post-partum. Hence, gap in knowledge and practice to assess for quality of care for the population could not be determined. Another potential limitation is the cross-sectional nature of the survey which does not allow us to infer causality. We did not have information on important potential confounders like distance and functionality of nearby health facility and could not adjust for them. Further, this survey covered section of the population predominantly belonging to SC/ST and other backward classes, hence generalizing the findings to a larger population would be debatable.

## Conclusion

In conclusion, the baseline survey showed lack of knowledge of maternal and newborn care and poor practice of healthy behaviors among women and a few statistically significant associations with intensity of contact with FLW. The results brought to light the fact that contact with FLW does not necessarily bring about a behavioral change in people; probably quality of services provided by FLW and timings of contact may be crucial. Building a sustainable impact on maternal and newborn health might require moving beyond FLWs (as just the agents of change) to community mobilization through platforms like SHGs. Such participatory approach should be taken advantaged of to provide a way forward for more scalable strategies. However, these findings need to be interpreted in the context of the organizational and programmatic aspects and functionality of the health system keeping in mind that FLW and/or SHG alone cannot bring about the desired reduction in maternal and child mortality.

## Additional file

Additional file 1: Box S1. Overview of administrative and healthcare system in India, frontline workers under government health system and Rajiv Gandhi Mahila Vikas Pariyojana (RGMVP) (DOCX 15 kb)

## Abbreviations

ANC: Antenatal care
ANM: Auxiliary nurse midwives
ASHA: Accredited social health activist
AWW: Anganwadi worker
BCC: Behavior change communication
BCM: Behavior change management
CAPI: Computer-assisted personal interview
CSPro: Census and survey processing system
FLW: Frontline worker
GP: Gram Panchayat
IFA: Iron-folic acid
IMR: Infant mortality rate
KMC: Kangaroo Mother Care
MDG: Millennium Development Goal
MLE: Monitoring learning and evaluation
MMR: Maternal mortality ratio
NMR: Neonatal mortality rate
PNC: Postnatal care
RGMVP: Rajiv Gandhi Mahila Vikas Pariyojana
RMNCHN: Reproductive, Maternal, Newborn and Child Health and Nutrition
SD: Standard deviation
SDG: Sustainable development goal
SHG: Self-help groups
SS: Swasthya Sakhi
TT: Tetanus toxoid
UP: Uttar Pradesh
UPCM: Uttar Pradesh Community Mobilization
