# Supplementary material for: Intensity of contact with frontline workers and its influence on maternal and newborn health behaviors: cross-sectional survey in rural Uttar Pradesh, India
Source: J Health Popul Nutr. 2018 Jan 8;37:2. doi: 10.1186/s41043-017-0129-6 (PMC5759258; doi:10.1186/s41043-017-0129-6)
Supplement: Additional file 1: Box S1. — Overview of administrative and healthcare system in India, frontline workers under government health system and Rajiv Gandhi Mahila Vikas Pariyojana (RGMVP) (DOCX 15 kb) [file 41043_2017_129_MOESM1_ESM.docx]

**Box S1: Overview of administrative and healthcare system in India, frontline workers under government health system and Rajiv Gandhi Mahila Vikas Pariyojana (RGMVP)**

**Administrative division**: India is a large country comprising of 28 states and 7 union territories. These states and the union territories are divided into districts. Each district is further divided in to sub-districts, which are known differently in different parts in the country (e.g., tehsil, taluka, community development (CD) block, Police station, Mandal, revenue circle, etc.). The lowest primary administrative units of administration are the villages in rural areas and towns in urban areas. All the villages and towns usually form part of a sub – district. While urban areas of the districts are organized further into town area committees, municipal boards and corporations, the rural areas have a three-tier structure of local self-governments called panchayati raj with Gram Panchayats existing at the village level.

**Healthcare delivery system in India**: There are three levels of healthcare delivery in India. Primary healthcare, the first level of contact between communities with the health system, is provided through a network of Sub centres and Primary Health Centres (PHC) in rural areas, and through Health posts and Family Welfare Centres in urban areas. The Sub centre consists of one Auxiliary Nurse Midwife and Multipurpose Health worker and serves a population of 5000 in plains and 3000 persons in hilly and tribal areas. The Primary Health Centre (PHC), staffed by Medical Officer and other paramedical staff serves every 30000 population in the plains and 20,000 persons in hilly, tribal and backward areas. Each PHC is to supervise 6 Sub centres. Secondary Healthcare refers to a second tier of health system, in which patients from primary health care are referred to specialists in higher hospitals for treatment and they include District hospitals and Community Health Centre at block level. Tertiary Health care refers to a third level of health system, in which specialized consultative care like Intensive Care Units, advanced diagnostic support services and specialized medical personnel are provided. Under the public health system, tertiary care service is provided by medical colleges and advanced medical research institutes.

**Frontline workers (FLWs) under Indian health system**: In the Indian healthcare system, the role of FLWs became prominent with the Alma Ata Declaration in 1978 that acknowledged primary health care as the key element for improving community health. They are members selected from the community and are supported by the health system. They function as service extension workers and as activists for social change. As service extension workers, they support the different health programmes in rendering services such as immunisation and health promotion. As social activists, they act as intermediaries to strengthen the interface between the health system and the community through facilitating community participation and involvement. While Auxiliary Nurse Midwives (ANMs) and Accredited Social Health Activists (ASHAs) function are under the Ministry of Health, the anganwadi workers (AWWs) are affiliated to the Ministry of Women and Child Development. The FLW undergoes extensive training to dispense their roles which mainly cover preventive aspects of maternal, new-born and child care. The FLWs (in particular, the ASHAs) are also incentivised under specific programs to promote maternal care i.e. promote institutional delivery and conduct postnatal care in the community. FLW are linked to government health facilities where they refer pregnant woman, new-born and children for care.

**Rajiv Gandhi Mahila Vikas Pariyojana (RGMVP):** The RGMVP is a flagship poverty alleviation programme of the Rajiv Gandhi Charitable Trust (RGCT), initiated in Uttar Pradesh (UP) in 2002 focusing on social capital building and empowerment of poor women through Self-Help Group (SHG) institutions. RGMVP’s SHGs consists of 10–20 poor women from poor socio-economic backgrounds. The programme utilizes community health volunteers (called Swasthya Sakhi, SS) who is a member selected from each SHG. SHGs are comprised of 10-12 women from rural households that are thought to be marginalised in terms of poverty, literacy, belonging to certain caste (scheduled) etc. Women become members of SHGs of their own will and are not paid any emoluments to be a member. Within SHG groups, women either volunteer to support the health promotion work as Swathya Sakhis or are nominated. Typically women who are vocal, energetic, have some level of literacy and are interested in health are chosen as SS.
